# Appraisal of primary immunodeficiencies in Latin America and the Caribbean using the IPOPI PID Life Index – where are we now?

**DOI:** 10.3389/fimmu.2026.1922796

**Published:** 2026-09-24

**Authors:** Leire Solis, Roberta Anido de Pena, Adli Ali, Antonio Condino-Neto, Nizar Mahlaoui, Lorena Regairaz, Silvia Sánchez-Ramón, Carolina Prando, Johan Prevot, Martine Pergent, Maria Cecilia Poli

**Affiliations:** 1 International Patient Organisation for Primary Immunodeficiencies (IPOPI), Brussels, Belgium; 2 Asociación de Ayuda al Paciente con Inmunodeficiencia Primaria (AAPIDP), Buenos Aires, Argentina; 3 Department of Paediatrics, Faculty of Medicine, University Kebangsaan Malaysia, Kuala Lumpur, Malaysia; 4 Clinical Immunology, Hospital Tunku Ampuan Besar Tuanku Aishah Rohani, University Kebangsaan Malaysia (UKM) Specialist Children’s Hospital, Universiti Kebangsaan Malaysia, Kuala Lumpur, Malaysia; 5 Senior Professor of Immunology, Institute of Biomedical Sciences, University of Sao Paulo, Sao Paulo, SP, Brazil; 6 French National Reference Center for Primary Immune Deficiencies (CEREDIH), Necker Enfants Malades University Hospital, Assistance Publique-Hôpitaux de Paris (APHP), Paris, France; 7 Pediatric Immuno-Haematology and Rheumatology Unit, Necker Enfants Malades University Hospital, Assistance Publique-Hôpitaux de Paris (APHP), Paris, France; 8 Immunology Unit, Children´s Hospital Sor Maria Ludovica, Buenos Aires, Argentina; 9 Department of Immunology, Faculty of Medicine, Universidad Nacional La Plata, Buenos Aires, Argentina; 10 Department of Clinical Immunology, Institute of Laboratory Medicine and IdISSC, Hospital Clínico San Carlos, Madrid, Spain; 11 Department of Immunology, Ophthalmology and Otolaryngology (ENT), School of Medicine, Complutense University, Madrid, Spain; 12 Faculdades Pequeno Príncipe, Curitiba, PR, Brazil; 13 Hospital Pequeno Príncipe, Curitiba, PR, Brazil; 14 Instituto de Pesquisa Pelé Pequeno Príncipe, Curitiba, PR, Brazil; 15 Programa de Immunogenética e Imunología Translacional, Instituto de Ciencias e Innovación en Medicina, Unidad de Inmunologia y Reumatologia, Facultad de Medicina Clínica Alemana- Universidad del Desarrollo, Santiago, Chile; 16 Unidad de Inmunologia y Reumatologia, Hospital Roberto del Rio, Santiago, Chile

**Keywords:** Caribbean, diagnosis, Latin America, PID patient organisations, primary immunodeficiency, registries, specialised centres, treatment

## Abstract

**Objective:**

Analyse the implementation of the 6 Principles of Care (PoC) for primary immunodeficiencies (PIDs) in Latin America and the Caribbean countries.

**Methodology:**

Data was extracted from the IPOPI PID Life Index, an interactive tool measuring how it is to live with a PID in the 13 countries in Latin America and the Caribbean (Argentina, Bolivia, Brazil, Chile, Ecuador, El Salvador, Honduras, Mexico, Paraguay, Peru, Puerto Rico, Uruguay and Venezuela).

**Results:**

Care for PIDs in the region varies greatly. Low diagnostic rates and limited diagnostic test options are a reality for the region. Almost all countries provide certain therapies for the treatment of PIDs, mostly anti-infectious therapies, immunoglobulin replacement therapies and vaccines. Biological therapies and curative treatments are less frequent in the countries covered. Universal health coverage for people living with PIDs remains heterogeneous and incomplete. Specialised centres and registries are also heterogeneously implemented. National organisations representing patients with PIDs exist in almost all countries, mainly focussing on general and medical awareness raising, and patient support through social activities.

**Discussion and conclusion:**

PID care in Latin America and the Caribbean is steadily becoming a reality for many people living with PID. There have been significant improvements in terms of diagnosis, treatment and care in the region over the past decades. Still, there are significant inequalities across the different countries for the 6 PoCs for PID analysed. Joint efforts of medical specialists and patient organizations must continue to ensure an optimal environment for people living with PIDs in the region.

## 1 Introduction

Primary immunodeficiencies or (PIDs) are a group of 559 currently identified disorders (1, 2) in which the immune system does not function properly or at all. PIDs are generally considered rare diseases (3) and collectively affect 1 in 2,000 individuals. However, the prevalence may vary from country to country due to multiple factors. Most PIDs are caused by genetic defects of the immune system, which are often hereditary. People living with PIDs are more susceptible to infections, inflammation, autoimmunity, severe allergies, and malignancies, amongst other complications. When left undiagnosed or misdiagnosed, PIDs may lead to chronic illness, disability, permanent organ damage or even death (4).

Despite significant advances in the diagnosis of PIDs, a large proportion of affected individuals remain undiagnosed. Once diagnosed, a range of treatments is available depending on the specific condition. Depending on the type of PID, specialists may prescribe anti-infectious therapies, vaccines, immunoglobulin replacement therapies, biological therapies and curative options. The cost of a late diagnosis frequently results in a heavy disease burden on the patient. Evidence from Mexico documented how timely diagnosis and treatment of patients with PID with immunoglobulin replacement therapy significantly decreased the per-capita costs of healthcare by around 47% (5).

IPOPI is a non-profit international organisation dedicated to improving the quality of life for people with PID and associated conditions by raising awareness, promoting equitable access to early diagnosis, personalised treatment through global collaborations, partnerships and innovative solutions. As part of its activities, IPOPI convened a regional meeting with its National Member Organisations from Latin America in Buenos Aires (Argentina) on 16 and 17 October 2025. Held back-to-back with the LASID regional meeting, the event aimed to enhance and strengthen the network of patient advocates on issues related to access to care and early diagnosis in the region. Patient representatives discussed their national contexts, identified regional priorities, and agreed on recommendations and lines of action. IPOPI considered it important to assess the status of PID care in Latin America and initiated the development of this publication. To structure the analysis, the PID Life Index was selected.

The PID Life Index (‘Ipopi - PID Life Index’, n.d.) is an interactive tool developed by IPOPI in collaboration with an expert task force to measure the implementation of the PID Principles of Care worldwide. These Principles of Care, and therefore, the PID Life Index, are based on the following six principles: i) availability of PID diagnosis; ii) availability of treatments; iii) universal health coverage; iv) specialised centres; v) national patient organisations; vi) registries. Each of the principles is structured around a series of criteria and sub-criteria. Recently, reports describing the situation of the implementation of the PID Principles of Care worldwide (4) in 2022 and in South-East Asia (6) in 2024 have already been published. This publication is the first description of the Latin American region.

Latin America and the Caribbean comprise 33 countries and several territories (including Puerto Rico, Sint Maarten, the Virgin Islands and Saint Martin) that are not responsible for the conduct of international relations. The region represents approximately 8.1% of the world’s population (662 million inhabitants in 2024 (7)), including around 50 million Indigenous people belonging to 500 different ethnic groups. According to the Organisation for Economic Co-Operation and Development (OECD), health systems in the region face multiple challenges, including high levels of poverty, income inequality, labour informality, large populations living in informal settlements without access to essential services, and growing environmental threats that disproportionately affect vulnerable populations (8). In 2020, 16% of urban households and 21% of rural households did not have access to healthcare services when needed (7).

The shortage of PID-specialised physicians and nurses represents a clear bottleneck in healthcare delivery. Although healthcare expenditure relative to GDP has increased in recent years, it remains insufficient and relies excessively on private spending (8). Aligned with Sustainable Development Goal 3.8, the World Health Organization defines Universal Health Coverage (UHC) as ensuring all people have access to quality essential healthcare, safe and affordable medicines, and vaccines, all while maintaining financial risk protection (7). Between 2010 and 2019, health expenditure per person in the region grew faster than the average economic growth. Approximately 1.7% of the population became poor because of out-of-pocket health spending, and 12.7% of the population was pushed further below the poverty line (8).

Within this broader context, living with a PID in Latin America exacerbates existing challenges related to accessing a diagnosis, obtaining prescribed disease-modifying treatments (including lifesaving therapies and curative therapies), and ensuring sustained access to care for individuals. These challenges therefore have implications not only for individual health outcomes but also for healthcare resource utilisation, household financial burden and the sustainability and equity of health systems. Increasing awareness and knowledge of the PID Principles of Care to ensure adequate care for people living with PIDs is therefore of paramount importance. To address a critical gap in regional data, this article describes the current state of PID care in Latin America and the Caribbean, drawing on shared experiences from the 2025 IPOPI Latin American regional meeting in 2025.

## 2 Materials and methods

The analysis is based on the data extracted from the PID Life Index database on the basis of a survey used to build the PID Life Index. Further information on the development of the survey has been described in **Table 1**, based on previous publications (9), and was complemented with the exchanges held with patient representatives from Latin American countries attending the IPOPI Latin American regional meeting held in Buenos Aires (Argentina) in October 2025.

**Table 1:** Principles and description of the PoC used to build the PID Life Index.

Principle	Description
PID diagnosis	Availability of diagnostic tests for the diagnosis of patients with PIDs, as well diagnosis rate (percentage based on the theoretical number of PID patients and the known number of patients in that country).
Treatments	This principle considers the availability of treatments for people living with PIDs in a given country as well as the blood and plasma collection infrastructure for the development of plasma-derived medicines. A therapy is considered “available” if it has received a market authorisation and is present or can be accessed by patients with PIDs in the country, irrespective of whether patients need to pay for it or if it is covered.
Universal health coverage	Reimbursement or coverage (expressed in percentage) of a certain diagnostic test or a treatment for patients with PIDs living in a country.
Specialised centres	This principle details the availability of centres providing for children and adult patients with PIDs specialised care, including diagnosis, treatment, care and transition to adult services. The best care model is considered to be centres structured within a single national network.
National patient organisations	The presence of a national organisation representing both children and adults living with a PID is crucial. These national patient organisations offer invaluable support to individuals affected by PIDs, while also playing an active role in raising awareness, educating the public about the condition, and advocating for policy changes that better address the needs of their members.
Registries	Registries represent an essential tool to increase knowledge and understanding of rare diseases, as well as support for scientific and clinical research. This principle measures the existence of a register for PIDs in the country and of a bone marrow donor registry.

Countries were included in the present analysis when a completed survey was available from either an IPOPI National Member Organisation (NMO) or, in countries with no NMO, an IPOPI medical contact. Based on this, data were available from 13 countries out of the 33 countries/territories encompassing the Latin American region: Argentina, Bolivia, Brazil, Chile, Ecuador, El Salvador, Honduras, Mexico, Paraguay, Peru, Puerto Rico, Uruguay and Venezuela. Data for these countries were collected between 2020 and 2025 via a survey completed by the relevant IPOPI NMO or IPOPI medical contact, to the best of their knowledge and with the help of the organisation’s medical advisors. The survey responses were subsequently compiled in the PID Life Index database and constituted the dataset analysed in this study.

## 3 Results

### 3.1 Principle 1: availability of diagnosis for PID

The principle of diagnosis for PIDs is based on four sub-criteria: the availability of biological tests, genetic diagnosis, prenatal diagnosis and newborn screening, as described in **Figure 1**. According to the data provided by the national PID patient organisations, most countries report some level of diagnostic capacity. The most locally available test is biological diagnosis, with 10 countries providing it either fully (4 out of 13) or partially (6 out of 13); followed by genetic diagnosis, which is available in 9 countries (3 out of 13 fully and 6 partially). Prenatal diagnosis, a standard prenatal screening used when the family already has a child affected with a primary immunodeficiency with a known genetic cause, is not a common diagnostic tool in the region, as only 4 countries report partial or full availability.

**Figure 1:**
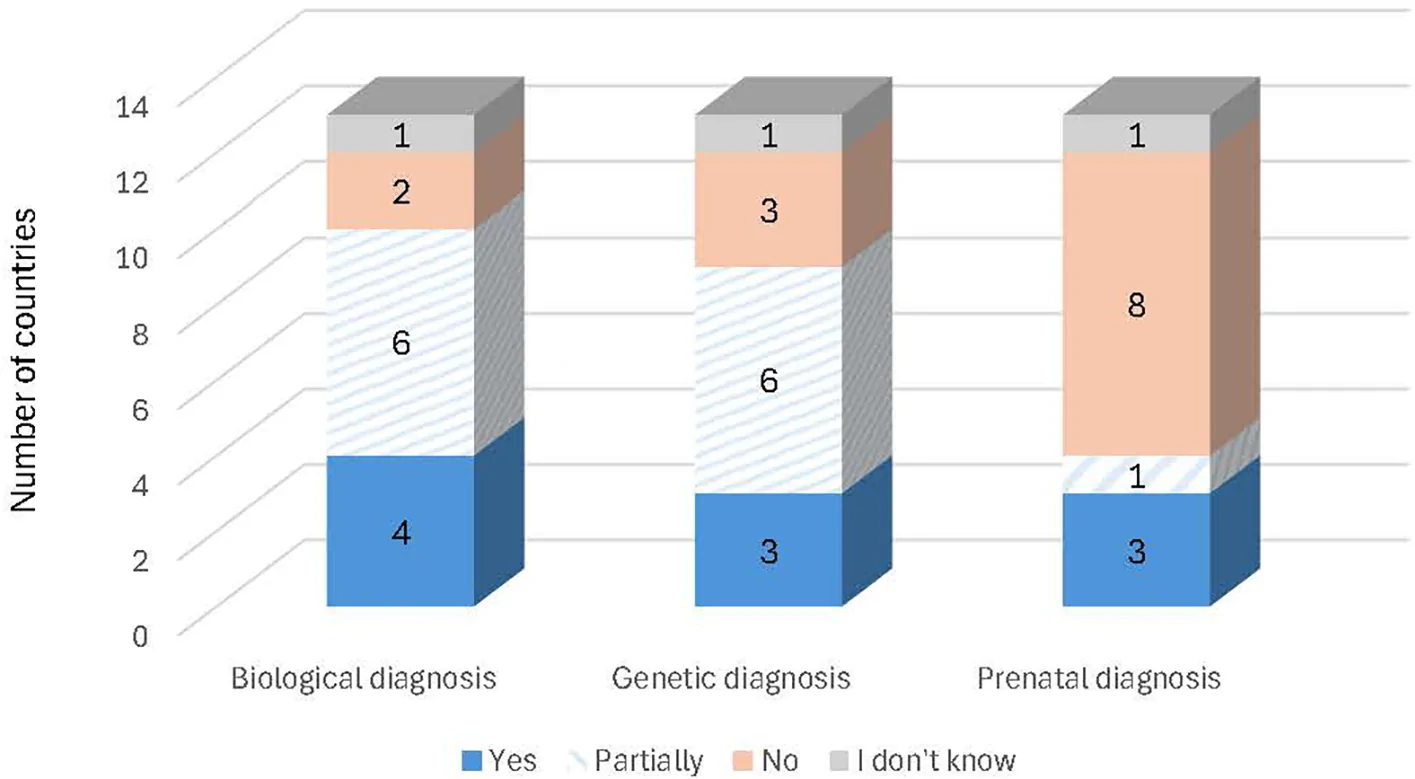
Diagnostic tests availability in the different countries (n=13).

The high underdiagnosis of PIDs in the region is partly due to the lack of availability of diagnostic tools, including the availability of genetic diagnosis to improve the accuracy of the PID diagnosis. The prevalence of PIDs varies greatly between regions depending on multiple factors, such as structural constraints (limited resources supporting healthcare professionals, expert centres for children and adults, registries, and diagnostic tools), as well as cultural and geographical factors (consanguinity, closed communities). The diagnosis rate in the Index represents the ratio of identified patients to the theoretical prevalence of PIDs, which is estimated at 1 in 2,000 inhabitants. Argentina reports the highest diagnosis rate, with 13% of theoretical cases diagnosed, followed by Uruguay (10%) and Puerto Rico (9%). On the other end of the spectrum, Honduras reports a diagnosis rate of 0%, with only 13 diagnosed people living with PIDs in a country with more than 11 million inhabitants in 2025. El Salvador did not provide data for this indicator and was therefore also assigned a diagnosis rate of 0%. These figures are significantly higher than those reported in the South-East Asian region (6), where Thailand reports the highest diagnosis rate, albeit at 1%.

#### 3.1.1 Newborn screening for severe combined immunodeficiencies

Newborn screening (NBS) for severe combined immunodeficiencies (SCID) is a crucial intervention that dramatically improves the quality of life and life expectancy of babies born with SCID (10, 11). Following the 2018 United States of America recommendation for nationwide SCID screening, numerous countries have integrated SCID into their national NBS panels. In Latin America, only Puerto Rico has fully implemented it, while Argentina and Brazil have launched regional programmes in recently, which shows some progress in this area. However, there are still 9 countries in the region that do not report any progress towards the national implementation of NBS (**Figure 2**).

**Figure 2:**
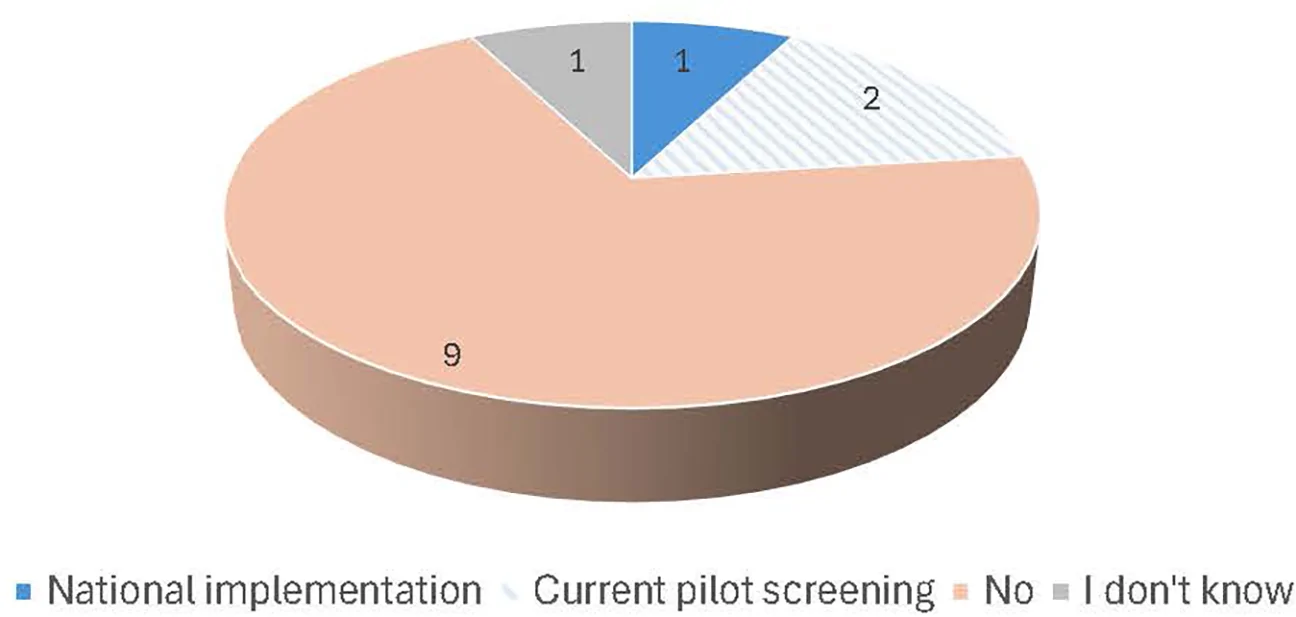
Number of countries having newborn screening for SCID (n=13).

### 3.2 Principle 2: availability of treatments for patients with PIDs

There is a wide range of therapies available for PIDs, as described in **Table 2**. These can be organised into two categories: supportive (curative or preventive anti-infective therapies, such as chemoprophylaxis, immunoglobulin replacement therapies, vaccines, and disease-modifying therapies such as biologicals or small molecules) or curative (hematopoietic stem cell transplantation [HSCT], gene therapy [GT], or thymic transplantation).

**Table 2:** Availability of treatments for patients with PIDs in Latin American countries.

Types of treatment	Availability of treatment (n=13)				
	No	Not regularly	Most of the time	Yes	I don't know
Anti-infectious					
Antibiotics	0	0	3	10	0
Antivirals	0	1	4	8	0
Antifungals	0	3	3	7	0
Antiparasitic	0	0	6	7	0
Immunoglobulins replacement therapy					
Intravenous immunoglobulin (IVIg)	0	2	2	9	0
Subcutaneous immunoglobulin (SClg)	5	4	0	4	0
Facilitated subcutaneous immunoglobulin (fSClg)	9	2	0	2	0
Biological treatments					
Enzyme replacement therapy	8	2	0	2	1
Immunosuppressors/modulators	0	4	3	4	2
Monocional antibodies	0	3	4	4	2
Cytokines / Interleukines	4	6	0	2	1
Growth factors	2	2	3	5	1
C1 inhibitor concentrate	5	3	2	0	3
Thrombopoietin receptor agonist	5	2	1	2	3
**Vaccines**	1	0	2	10	0
Curative treatments					
HSCT/BMT	5	n.a.	n.a.	8	0
Gene therapy	11	n.a.	n.a.	2	0
Thymic transplant	11	n.a.	n.a.	0	2

n.a., non applicable.

#### 3.2.1 Anti-infectious therapies

People living with PIDs are particularly susceptible to recurrent, atypical and/or severe infections, making anti-infectious therapies essential for disease management. These therapies include antibiotics, antivirals, antifungals, and antiparasitic agents, which may be administered prophylactically to prevent infections or on demand when infections occur.

Most countries in the region report having access to anti-infectious therapies. Antibiotics are the most widely available, either fully available or available most of the time in all countries surveyed. Antivirals are fully available in 8 out of 13 countries and available most of the time in an additional 3. Antiparasitic agents are fully available in 7 countries and available most of the time in the remaining 6, while antifungals are fully available in 7 out of 13 countries and available most of the time in 3. Several countries; namely Argentina, Bolivia, Chile, Peru, Puerto Rico, and Uruguay, report full availability of all categories of anti-infectious therapies.

#### 3.2.2 Immunoglobulin replacement therapies

Immunoglobulin replacement therapy (Ig) represents the cornerstone of treatment for a significant number of patients with PID (as it is estimated 50% to 60% of people living with PIDs present with a predominantly antibody deficiencies (12), not to mention patients with combined immunodeficiency who often present with some degree of antibody deficiency) (13). Immunoglobulin products are administered via two primary routes (the intramuscular one being out of use except in very rare cases): intravenous (IVIg) or subcutaneous, the latter of which includes both conventional (SCIg) and facilitated (fSCIg) methods (**Figure 3**). These therapies are typically prescribed lifelong and administered at regular intervals to maintain serum IgG levels sufficient to protect against bacterial and viral infections.

**Figure 3:**
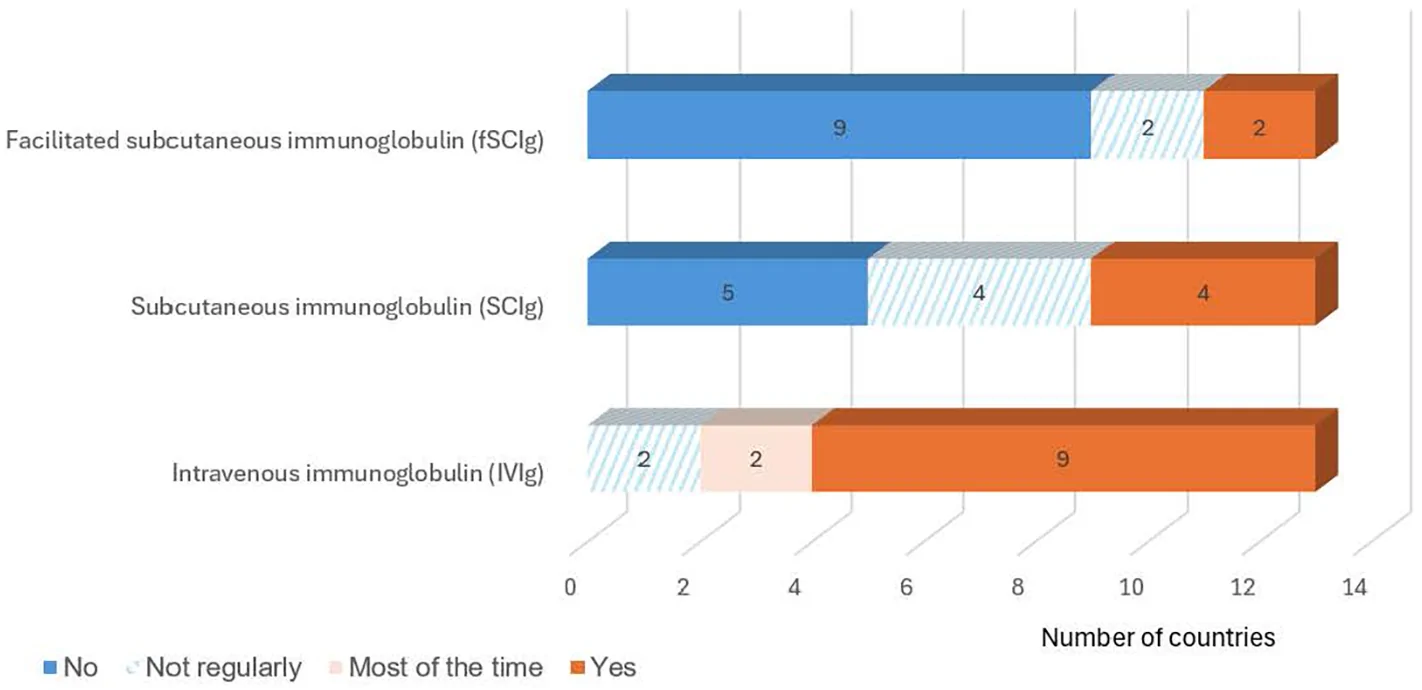
Immunoglobulin replacement therapies availability (n=13).

No single immunoglobulin product or route of administration meets the needs of all patients. Ensuring access to a range of products and administration routes is therefore critical to enable personalised treatment, optimise clinical outcomes, and support long-term adherence. The WHO recognises immunoglobulins as essential medicines for adults (14) and children with PIDs (15).

In Latin America, IVIg remains the most widely available route, with nine out of 13 countries reporting full access, two reporting access most of the time, and two reporting irregular access. Access to SCIg is more limited, with five countries reporting no availability and only four reporting full access. Availability is even more restricted for fSCIg (mode of administration in which the therapy is infused every 3–4 weeks subcutaneously), which is not available in 9 out of 13 countries and is fully available in only 2 countries. This distribution reflects a global trend in which IVIg remains the predominant route of administration. Each administration route offers its benefits and risks that are not the same for the different patients. In addition to this, it was shown during the COVID-19 pandemic that persons with PID receiving their Ig treatment at the hospital were impacted by access to, frequency and/or continuity of care (16). On the other hand, those patients receiving Ig at home would reduce their exposure to the virus during the pandemic (17). Making both routes available in a country with different concentrations remains a challenge in many countries in the region.

#### 3.2.3 Vaccines

Vaccines play a critical role in reducing the risk of infectious diseases by stimulating the body’s natural immune defences. However, people living with certain PIDs may not respond adequately to live-attenuated vaccines, and in some cases, the attenuated pathogen may revert to virulence. The World Health Organization considers immunisation a cornerstone of primary health care and an indisputable human right (18).

Within the region, only one country (El Salvador) reports no access to vaccines for people living with PIDs. Most countries (10 out of 13) report that vaccines are available for this population, highlighting relatively broad access compared with other therapeutic categories.

#### 3.2.4 Availability of biological treatments

Seven categories of biological therapies are assessed within the PID Life Index: thrombopoietin receptor agonists; C1 inhibitor concentrate; growth factors (G-CSF, GM-CSF, EPO); cytokines and interleukins (including interferon gamma, IL-2, and IL-7); monoclonal antibodies; immunosuppressants and immunomodulators; and enzyme replacement therapy for ADA-SCID.

Availability of biological therapies varies considerably across Latin America (**Figure 4**). Growth factors are the most widely available biological therapies, with five countries reporting full availability and three reporting availability most of the time. In contrast, enzyme replacement therapy for ADA-SCID is rarely available, with eight out of 13 countries reporting no access and only Argentina and Puerto Rico reporting consistent availability. This pattern mirrors global trends observed in earlier publications (4). A notable proportion of “I don’t know” responses was recorded for several biological therapies, reflecting both their relative novelty and their use in specific PID subtypes rather than across the broader patient population. This finding underscores the need for increased awareness and education regarding emerging therapeutic options.

**Figure 4:**
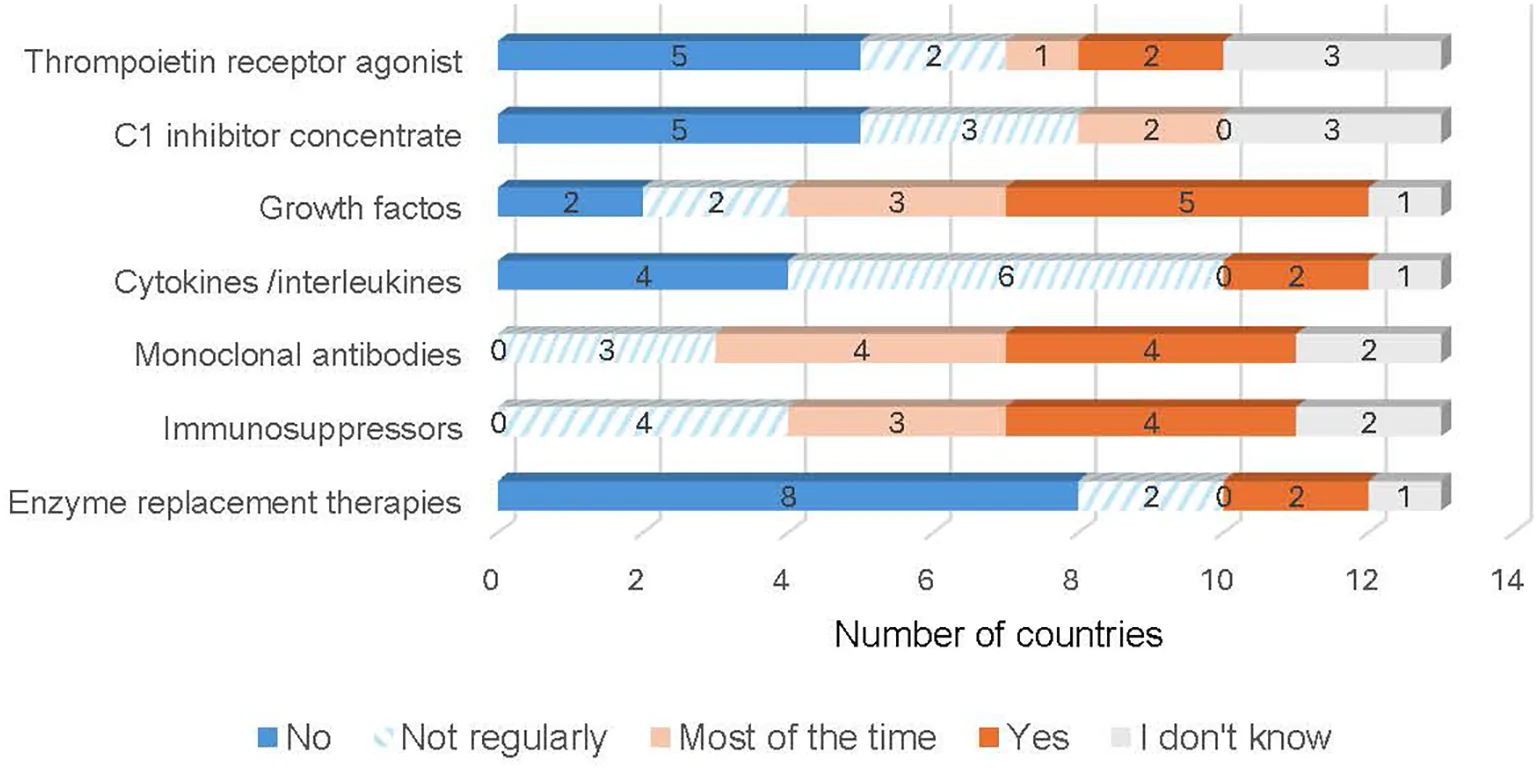
Availability of biological therapies (n=13).

#### 3.2.5 Curative treatments

Certain PIDs can be treated with interventions that offer the potential for long-term remission or cure. These curative options include HSCT, gene therapy, and thymic transplantation (**Figure 5**). Suitability varies by PID type and individual patient characteristics, requiring careful clinical evaluation ideally supported by genetic diagnosis.

**Figure 5:**
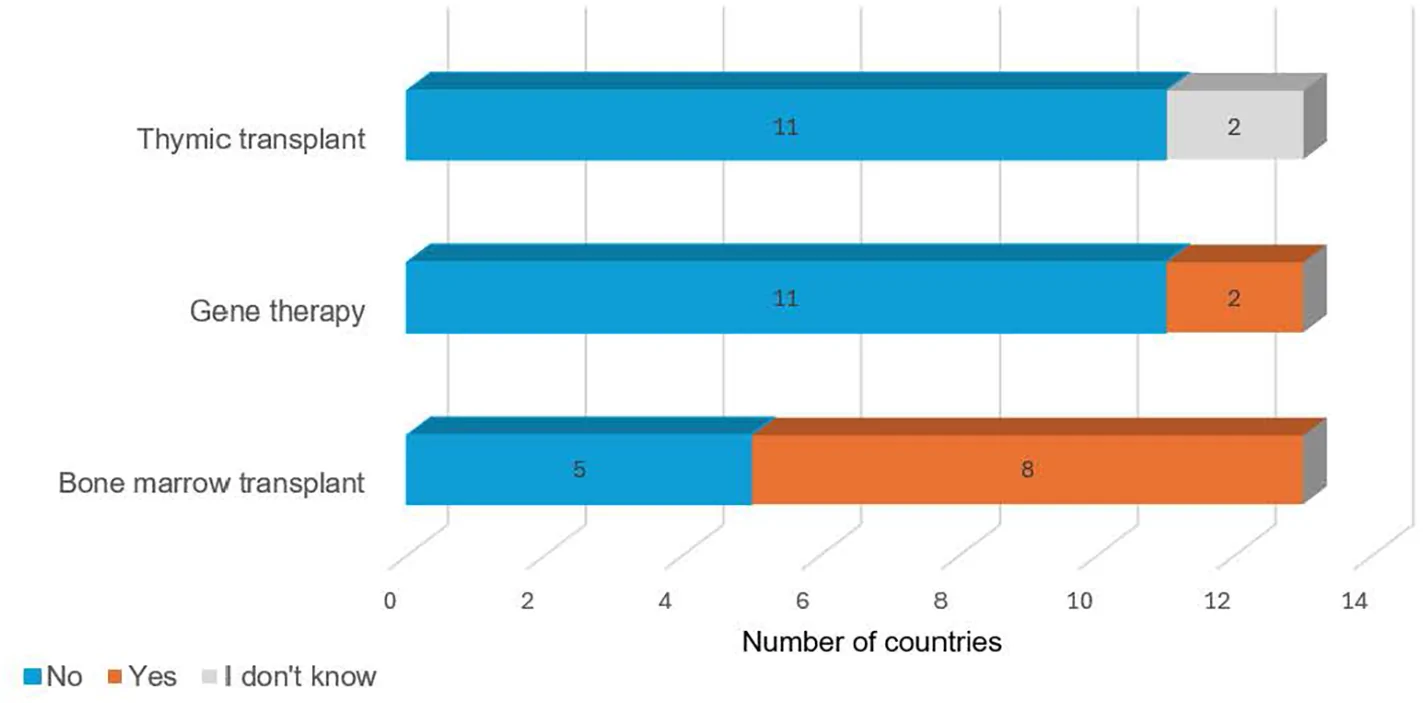
Availability of curative treatments (n=13).

In Latin America, HSCT and gene therapy (to a lesser extent) are the only curative treatments available (this will be further explained later). This has been described by a recent publication pulling data from the Latin American Society for Immunodeficiencies (LASID) Registry (19). HSCT is the most widely accessible option, with eight out of 13 countries reporting availability for people living with PIDs. Gene therapy is available in only two countries included in the PID Life Index. Thymic transplant/cultured thymic tissues are not available in 11 countries, while two countries reported uncertainty regarding their availability at the national level. Given that thymic transplant/cultured thymic tissues are performed in only a small number of specialised centres worldwide, limited awareness of this option within the region is unsurprising.

### 3.3 Principle 3: universal health coverage

Universal health coverage means that all people have access to the full range of quality health services they need, when and where they need them, without financial hardship (20). Importantly, the mere availability of diagnostic tools or therapies within a country does not guarantee that people living with PID can access them without incurring substantial out-of-pocket expenses. For chronic and rare conditions like PIDs, characterised by prolonged diagnostic journeys and lifelong management, comprehensive healthcare coverage is a critical determinant of patient outcomes. Crucially, such coverage is the only guarantee that prescribed therapies are administered in strict accordance with a physician’s specified frequency and dosage.

There is considerable variation across Latin American countries in the level of coverage provided for the diagnosis and treatment of people living with PIDs (**Figure 6**). Argentina stands out as the country offering the highest overall coverage at 68%, while El Salvador and Bolivia provide the lowest levels of coverage at 21% each. In countries with limited coverage, people living with PIDs and their families may be unable to afford diagnostic examinations or prescribed therapies, undermining their ability to achieve the recommended standard of care.

**Figure 6:**
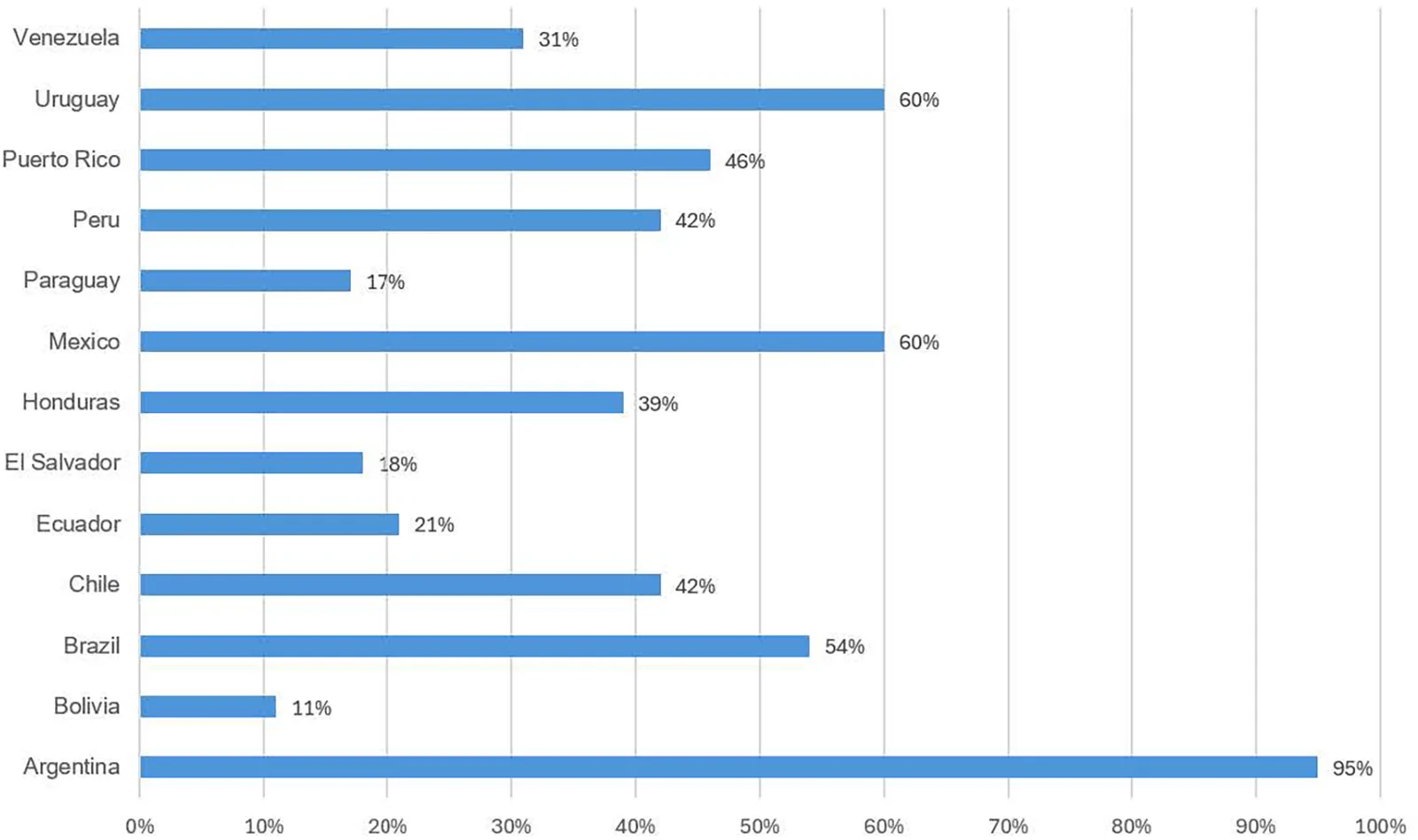
Scores of the 13 countries on the Principle Universal Health Coverage (in %).

#### 3.3.1 Coverage of diagnostic tests for PID

In most countries included in the PID Life Index, people living with PIDs or their families are required to fully cover the cost of genetic testing (eight out of 13 countries) and, although less available, for prenatal diagnosis (3 countries out of the 4 countries where it is available) (**Figure 7**). For biological diagnostic tests the situation is a bit different: in 4 countries patients or their families need to fully pay for them, in 4 other countries they are covered from 51% to 69% of their cost; in 2 countries they are fully covered and in 2 countries they are covered up to 50% or less. Argentina is the only country providing coverage for all three diagnostic modalities at levels exceeding 90%. Uruguay also offers partial coverage across all diagnostic categories, ranging from 51% to 69% for biological and prenatal testing and from 70% to 90% for genetic testing. In contrast, in Bolivia and Paraguay—where all three diagnostic options are available—the full cost must be borne by patients or their families. “Not applicable” was the option selected in those countries in which the diagnostic methods were not available for patients with PIDs and, therefore, could not be considered by coverage or reimbursement by the healthcare system or the patient’s insurance company.

**Figure 7:**
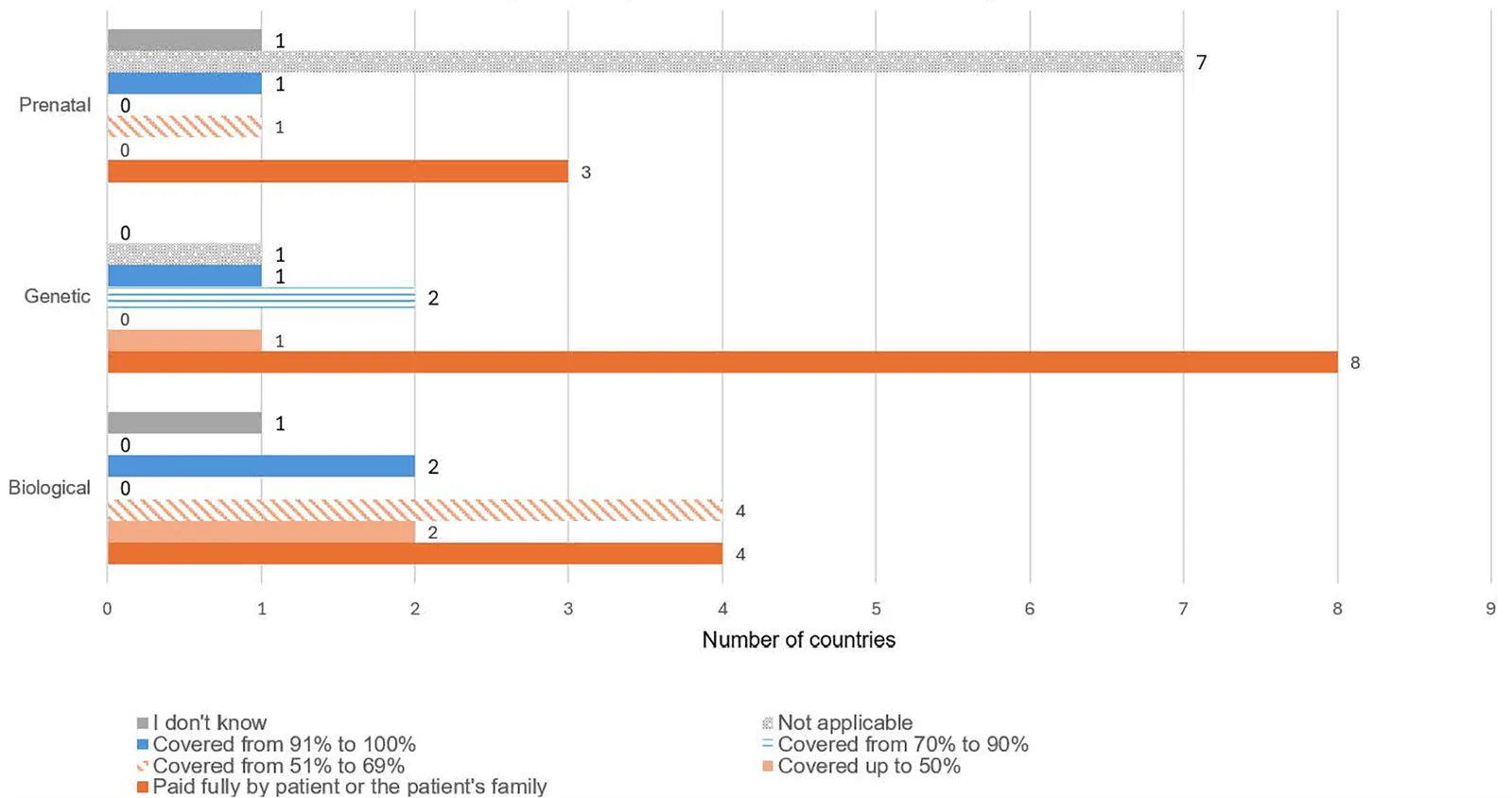
Coverage on diagnostic tests for PIDs (n=13).

#### 3.3.2 Coverage of anti-infectious therapies

Except for Bolivia, all countries in the region provide some degree of coverage for anti-infectious therapies for people living with PIDs (**Figure 8**). Compared with other therapeutic categories, coverage for anti-infectious treatments is relatively high. Antibiotics are covered at levels ranging from 91% to full coverage in five countries (Argentina, Brazil, Honduras, Mexico, and Uruguay). Antifungal therapies are covered at similar levels in four countries (Argentina, Brazil, Honduras, and Mexico) and up to 50% in four others (Chile, El Salvador, Peru, and Venezuela). Antiparasitic agents are covered at levels of 91% or higher in four countries, while antiviral therapies are mostly covered at levels between 70% and 90% in four countries (El Salvador, Paraguay, Puerto Rico, and Uruguay).

**Figure 8:**
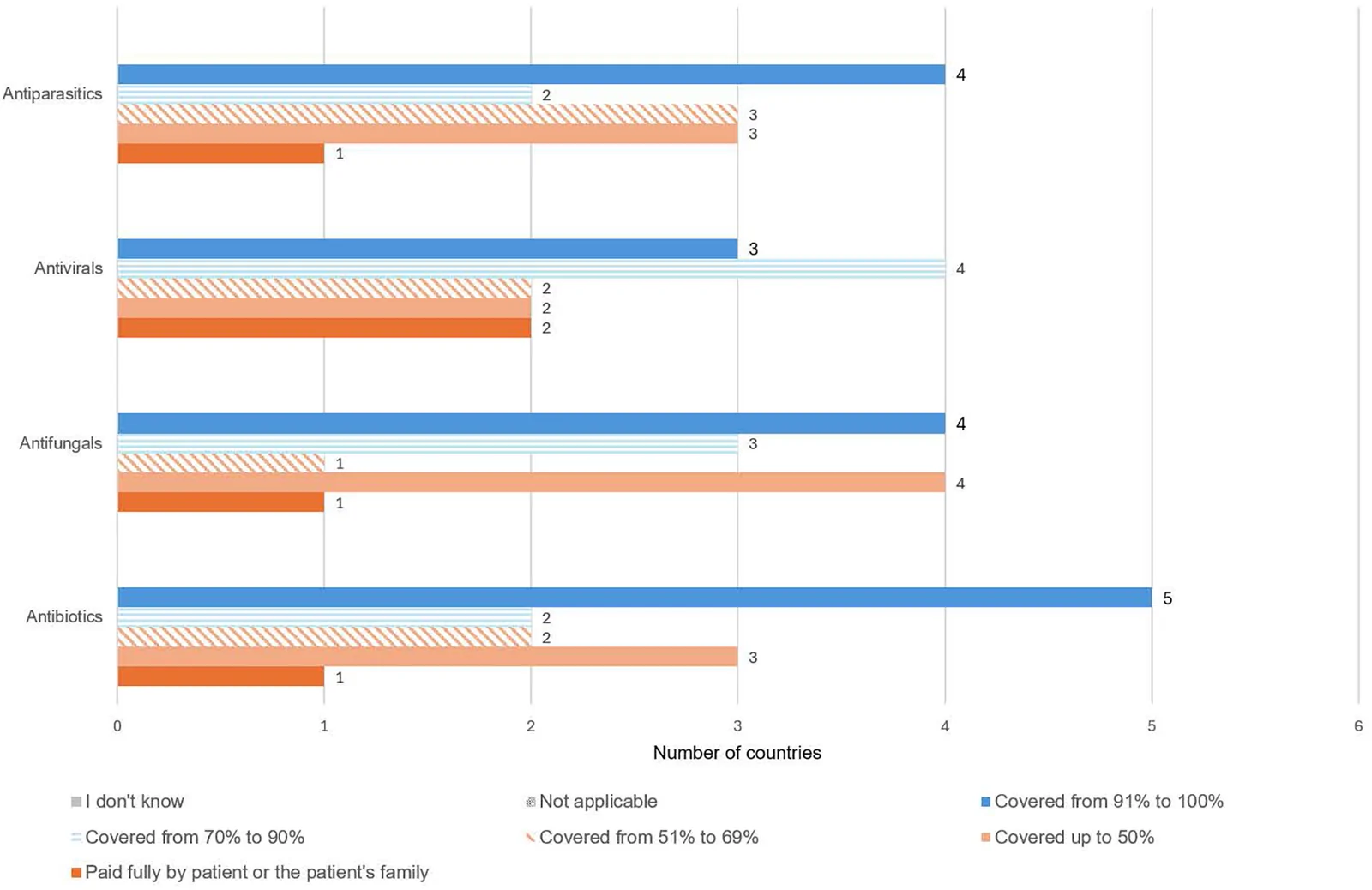
Coverage of anti infectious therapies for PIDs (n=13).

#### 3.3.3 Coverage of immunoglobulin replacement therapies

Immunoglobulin replacement therapies are required by a majority of people living with PIDs and are typically prescribed for life. As these therapies are significantly more costly than anti-infective treatments, reimbursement levels are a key determinant of access (**Figure 9**). IVIg is covered for diagnosed patients at levels ranging between 91% to full coverage in seven out of 13 countries (Argentina, Bolivia, Brazil, Chile, Honduras, Mexico, and Uruguay), while four countries (Ecuador, El Salvador, Puerto Rico, and Uruguay) provide coverage between 70% and 90%. SCIg is covered at levels exceeding 90% in four countries (Argentina, Chile, Mexico, and Peru) and between 70% and 90% in two countries (Puerto Rico and Uruguay). fSCIg is not fully covered in any country in the region, with only Argentina providing coverage between 70% and 90%. Despite incremental progress, substantial gaps remain, exposing many families to financial hardship.

**Figure 9:**
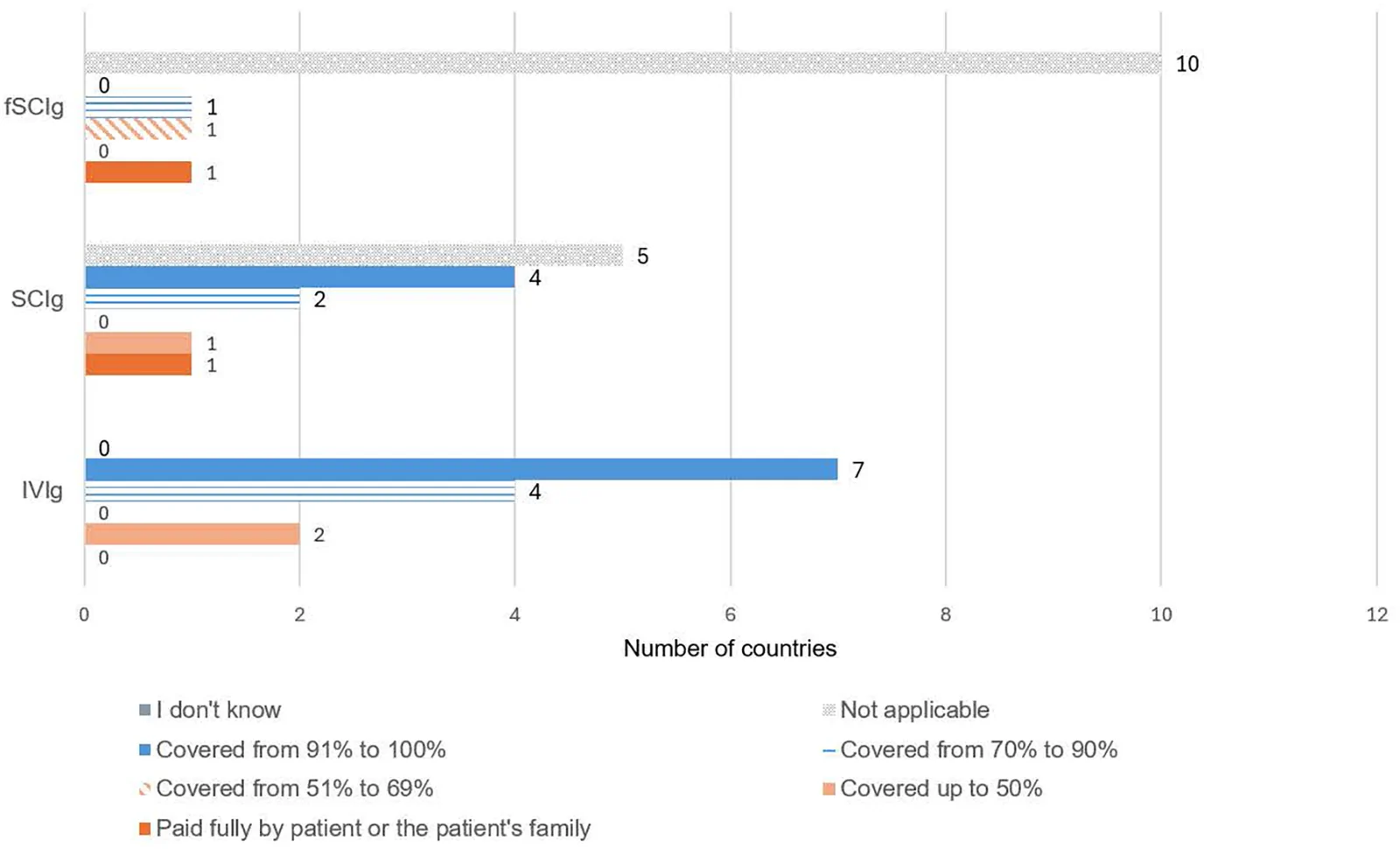
Coverage of immunoglobulin therapies for PIDs (n=13).

#### 3.3.4 Coverage of vaccines

All countries included in the PID Life Index provide some level of coverage for vaccines for people living with PIDs, when available. While Chile covers up to 50% of vaccine costs, three countries (Mexico, Puerto Rico, and Venezuela) provide coverage between 70% and 90%, and the remaining countries offer coverage levels exceeding 90% or full reimbursement.

#### 3.3.5 Coverage of curative treatments

HSCT is a complex procedure performed in expert centres, involving transplantation, hospitalisation, long-term follow-up, and associated indirect costs. In Paraguay, patients and families must fully cover the cost of HSCT, while Chile and Venezuela provide coverage of up to 50% (**Figure 10**). In contrast, five countries (Argentina, Brazil, Mexico, Peru, and Uruguay) cover between 91% and the full cost of the procedure. Gene therapy is available in only two countries; in Chile, the coverage of the procedure is not known, whereas in Puerto Rico coverage ranges from 70% to 90%.

**Figure 10:**
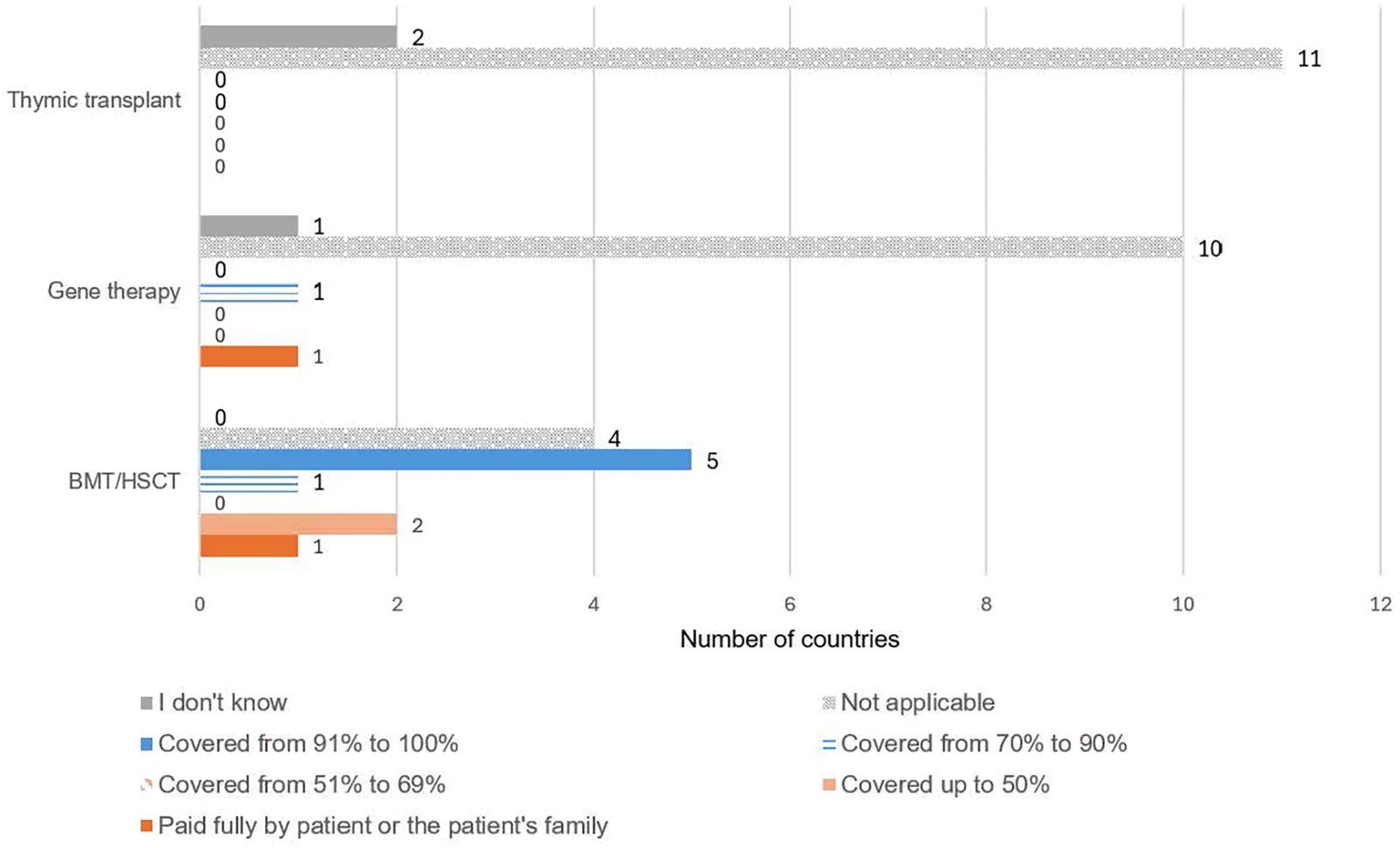
Coverage of curative treatments for PIDs (n=13).

#### 3.3.6 Coverage of biological treatments

Where biological therapies are available, most countries provide at least partial reimbursement (**Figure 11**). Growth factors are covered at levels exceeding 90% in five countries (Argentina, Honduras, Mexico, Uruguay, and Venezuela) and between 70% and 90% in Puerto Rico. Monoclonal antibodies and immunosuppressants or immunomodulators are covered at similar levels in four countries (Argentina, Honduras, Mexico, and Venezuela). In contrast, C1 inhibitor concentrates and thrombopoietin receptor agonists generally require full out-of-pocket payment. Limited awareness amongst patient organisations regarding reimbursement of certain biological therapies highlights ongoing gaps in knowledge and access.

**Figure 11:**
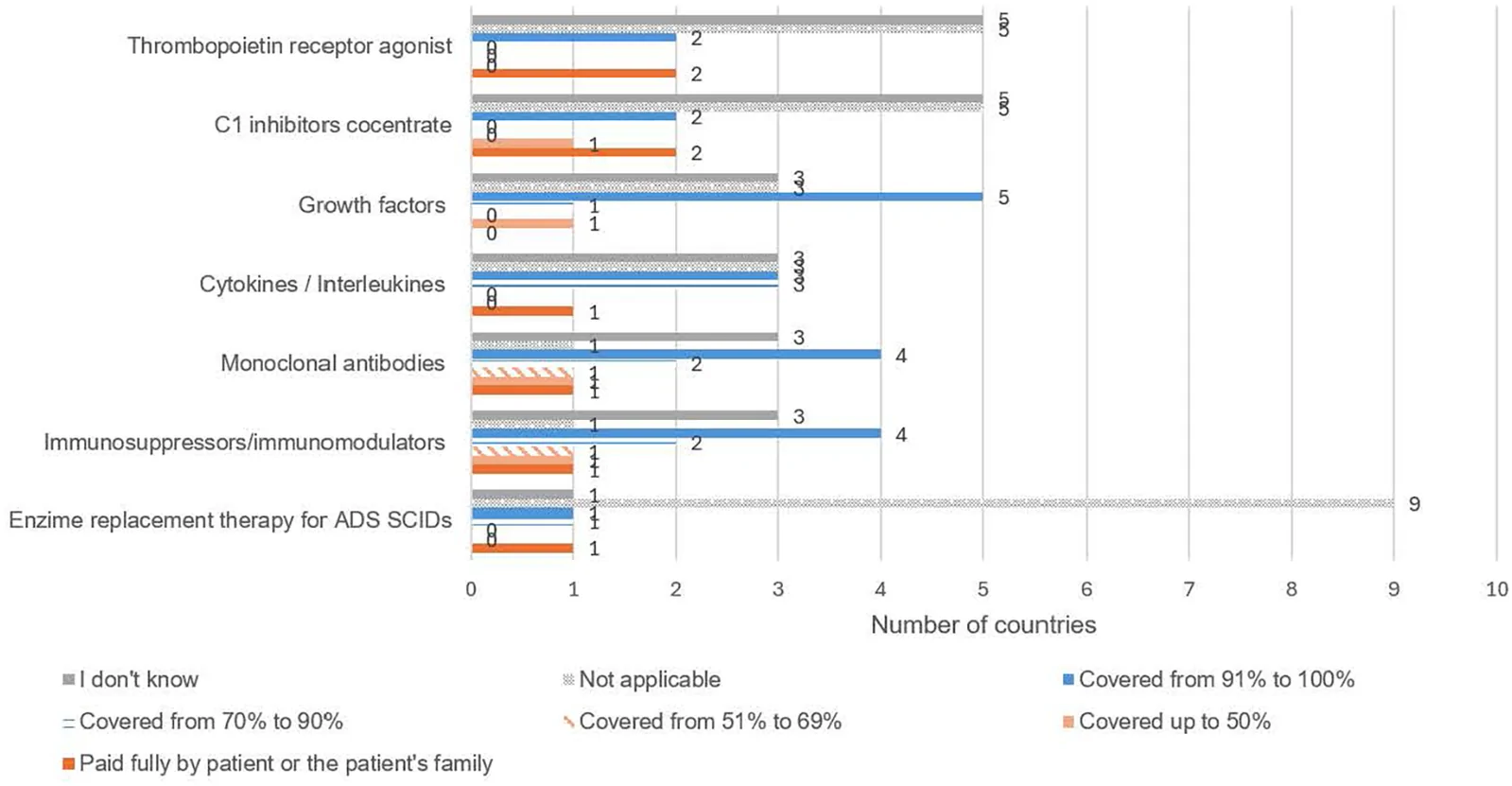
Coverage of biological therapies for PIDs (n=13).

#### 3.3.7 Overall universal health coverage in Latin America

Universal health coverage for people living with PIDs in Latin America remains heterogeneous and incomplete, reflecting structural health, social, and economic inequalities both between and within countries. While all countries included in the PID Life Index report provide some degree of coverage for diagnosis and treatment, the level of financial protection varies widely and often exposes people living with PIDs and their families to substantial out-of-pocket expenses.

Discussions during the IPOPI 2025 Latin American regional meeting highlighted that, in several countries, full coverage is more consistently granted during childhood following diagnosis but progressively declines sometimes during adolescence and in many when reaching adulthood. This reduction in coverage over time places additional strain on patients and families precisely at stages of life when continuity of treatment and long-term disease management are essential. Furthermore, even when patient rights are legally recognised, their enforcement often necessitates judicial intervention. In many cases, patients must resort to litigation to ensure these rights are fully respected and upheld.

The findings from the PID Life Index show that higher levels of coverage are more frequently achieved for anti-infectious therapies and vaccines, while diagnostic tests, immunoglobulin therapies, biologicals and curative therapies are less consistently reimbursed. This discrepancy undermines the core principle of Universal Health Coverage, which is rooted in the mandate that all individuals and families access necessary healthcare without experiencing catastrophic financial hardship.

### 3.4 Principle 4: specialised centres

People living with PIDs should be managed in specialised centres to ensure access to adequate medical and nursing expertise. While the structure and the capacity of these centres may vary according to national resources, available expertise and healthcare system; they should comply with internationally agreed standards of care and provide a holistic approach encompassing diagnosis, treatment, long-term follow-up and psychological support. In line with the PID Principles of Care, specialised centres should provide care for both children and adults; including structured transition services, specialised diagnosis and management services, engage in continuous training and professional development, contribute to PID-related research and promote effective integration with other hospital specialists to optimise patient outcomes and care. To reduce disparities in access and quality of care, these specialised centres should be interconnected within a coordinated national or regional network, supported by formal referral pathways for diagnosis, treatment, and follow-up. Such networks facilitate the sharing of clinical guidelines, patient registries, peer review, and professional leadership, thereby strengthening standards of care and ensuring that patients receive appropriate and timely PID care regardless of their place of residence (4).

#### 3.4.1 National PID specialised centres/network

None of the countries included in the PID Life Index has an established national network of specialised PID centres (**Figure 12**). Mexico and Puerto Rico report the existence of several uncoordinated networks. Five countries — Argentina, Brazil, Peru, Uruguay, and Venezuela — have multiple specialised centres providing diagnosis and care, while El Salvador and Paraguay each have a single specialised centre. Bolivia, Chile, Ecuador, and Honduras report no specialised PID centres. A national network of specialised PID centres has been considered as the benchmark for PID care organisation, as it allows for scientific and medical cooperation while ensuring national equity for patients in need, independently from where in the country they live on. However, PID specialists highlight the support provided by the Latin American Society for Immunodeficiencies (LASID) and associated institutions in offering opportunities for experts in the field to network and drive knowledge and care in the region.

**Figure 12:**
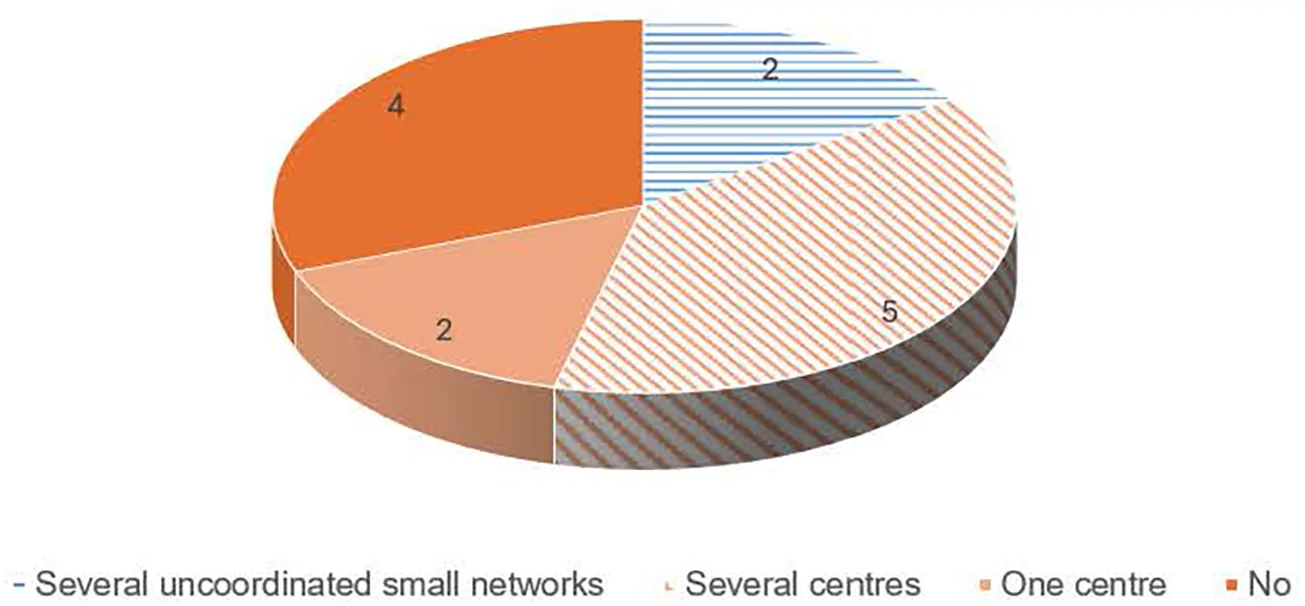
National PID specialised centre/network (n=13).

#### 3.4.2 Adult care

With improved diagnosis, treatment and care of persons with PIDs, as well as with increased knowledge and awareness of certain PIDs affecting adults, there is an increased number of adults living with a PID in the world.

Despite this trend, no country in the region has a coordinated national network for adult PID care (**Figure 13**). Mexico and Puerto Rico provide adult care through uncoordinated networks, while Argentina, Brazil, Peru, and Uruguay offer adult care within a single centre. Seven countries do not provide specialised adult PID care at all.

**Figure 13:**
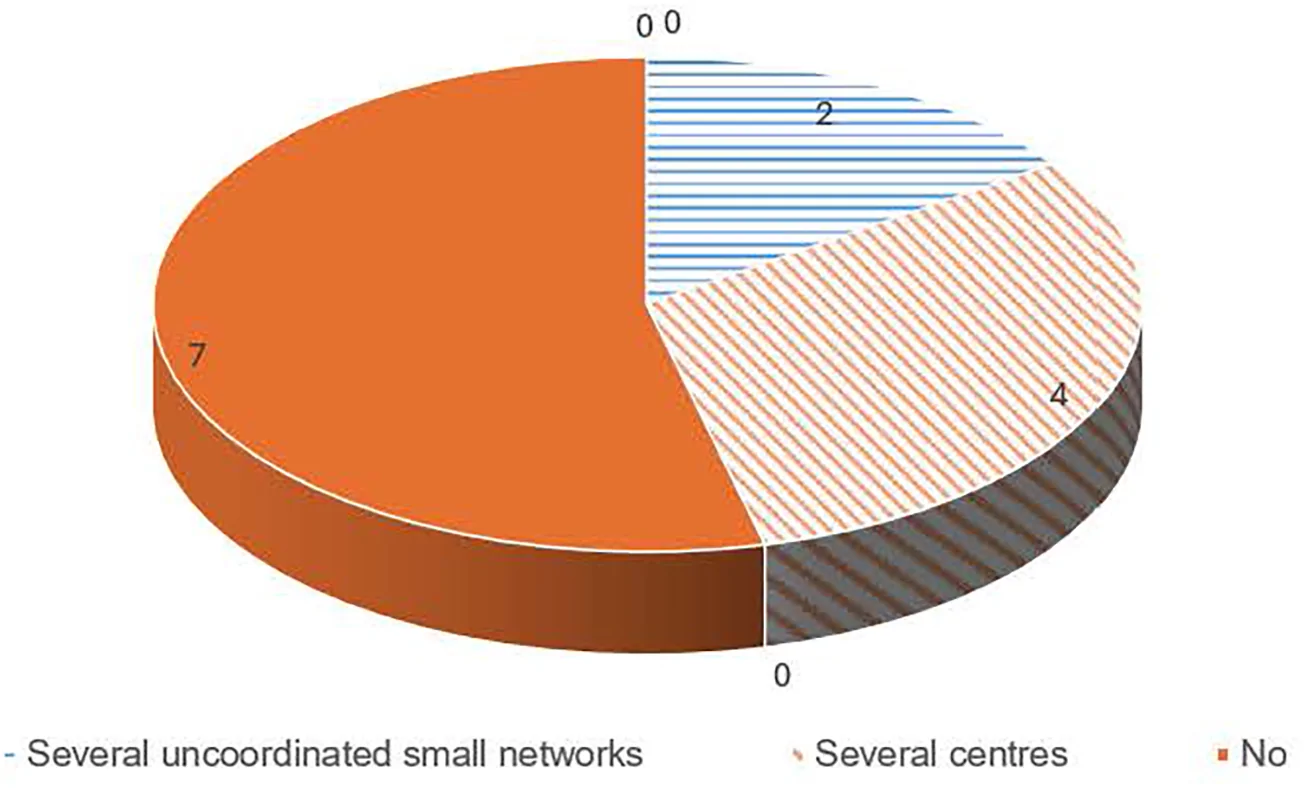
Adult PID services in specialised centres (n=13).

#### 3.4.3 Transition of care from paediatrics to the adult setting 

Many persons with PIDs are diagnosed in childhood and improved diagnosis, treatment and care for the different conditions have led to an increased life expectancy. This means that many more PID patients now transition from paediatric to adult healthcare services (21). Despite its importance, structured transition of care is non-existent in the vast majority of Latin American countries, with ten out of 13 nations lacking an official pathway for maturing PID patients (**Figure 14**). Only Argentina and Mexico have a transition of care programme in some specialised centres in the country. The absence of coordinated transition pathways represents a significant gap in health system preparedness and increases the risk of adolescents and young adults being lost to follow-up.

**Figure 14:**
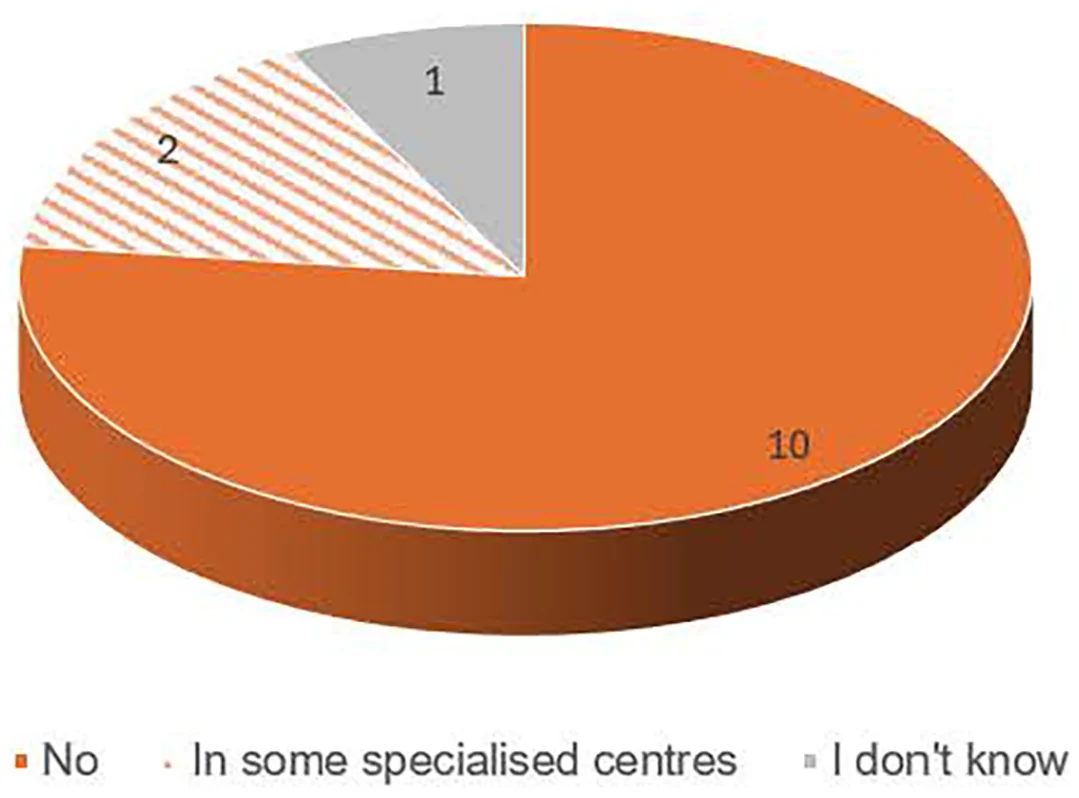
Transition of care of persons with PIDs (n=13).

In conclusion, although important gaps and disparities in the organisation of PID care remain across Latin America, notable progress has been achieved in recent years. Advances in scientific knowledge, increased awareness, and improvements in clinical management have contributed to better diagnosis and care for patients with PIDs in the region. Collaborative efforts, particularly through regional organisations such as LASID or medical networks as the Jeffrey Modell Foundation (JMF), have played a pivotal role in fostering cooperation amongst specialists, promoting education and training, and facilitating the exchange of expertise across countries. This growing network of committed professionals has helped to partially overcome structural limitations, driving forward both research and clinical practice.

Continued strengthening of these collaborative frameworks, alongside the development of coordinated national networks and structured care pathways, will be essential to consolidate these gains and ensure equitable, high-quality care for all individuals living with PIDs in the region.

### 3.5 Principle 5: national patient organisations

National patient organisations play a central role in representing people living with PIDs at country level. As IPOPI members, these organisations represent both paediatric and adult patients, operate nationally, and engage in advocacy, awareness raising, and patient education and support. Their role in health policy dialogue has become increasingly important, particularly in the field of rare diseases.

All countries included in the PID Life Index have one national patient organisation, with the exception of Honduras. Membership numbers vary widely, from 6 members in El Salvador to 426 in Argentina. Notably, the Argentinian organisation (Asociación Ayuda al Paciente con Inmunodeficiencia Primaria, AAPIDP) was the first to be established in the region. None of the organisations employ professional staff, relying entirely on volunteer contributions.

The scope of national patient organizations encompasses a broad spectrum of activities, ranging from foundational awareness and education to the promotion of clinical trials and research initiatives. From what we can see in **Table 3**, nearly all patient organisations (10 out of 11) work on the provision information and awareness raising about PIDs. Working on medical awareness is the second main area of work for the patient organisations (for 9 out of 11), highlighting the need to continue spreading awareness. Patient support through social activities is also an area of high interest amongst the patient organisations (8 out of 11). Seven out of 11 patient organizations in the region actively engage in medical professional cooperation, patient empowerment initiatives, and data collection.

**Table 3:** Main working areas of the national patient organisations (NMOs) in Latin America.

Main working areas of the national patient organisations (NMOs) in Latin America, n=11	
Awareness / information	10
Medical awareness	9
Patient support – social activities	8
Cooperation with medical professionals	7
Patient empowerment	7
Data collection	7
Patient support - psychological	5
Access to rights and legal support	5
Encouraging plasma/blood/bone marrow donations	3
Advocacy	3
Patient support – financial for access to medicines / care	3
Involvement in research / clinical trial promotion	2
Training – patient therapeutic education	2

This wide range of activities is developed with limited resources. At the IPOPI 2025 Latin American regional meeting, patient organisations representatives detailed the challenges their organisations face in recruiting volunteers (either patients or family members), especially amongst young people and with limited financial resources. The national patient organisations’ work was done, sometimes, in a context of political instability or political changes that prevented the swift implementation of the adopted political reforms or required new outreach to new politicians recently elected.

One main concern for national patient organisations in Latin America is the lack of diagnosis of adults with PIDs (**Figure 15**). The lack of patient diagnosis is a key concern for national patient organisations in Latin America. With very low diagnosis rates in the region, 6 national patient organisations are concerned about the diagnosis of adults with PIDs (Bolivia, Chile, El Salvador, Peru, Uruguay and Venezuela) and 5 are concerned about the diagnosis of children with PIDs (Bolivia, Brazil, Chile, El Salvador and Venezuela). Transition care from childhood to adulthood is an issue for 4 countries (Argentina, El Salvador, Peru and Venezuela), showing an increased interest from patient organisations to ensure that those patients diagnosed in childhood have a right transition of care to the adult specialists and are able to cope with the changes inherent to this healthcare system change. Treatment safety issues is an area of concern highlighted by 3 national patient organisations (Brazil, Chile and Peru).

**Figure 15:**
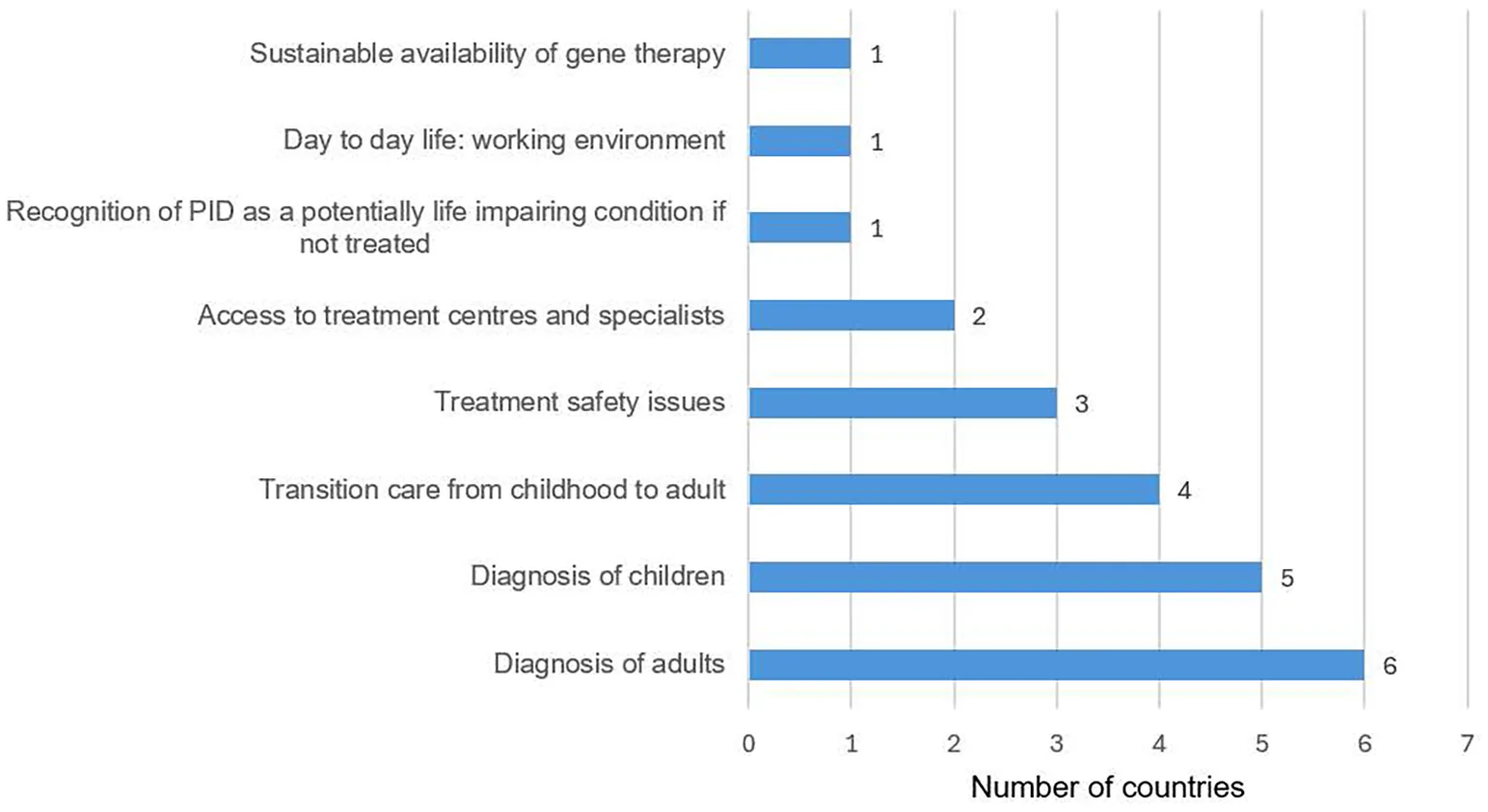
Main areas of concerns of national PID patient organisations in Latin America (n=11).

### 3.6 Principle 6: registries

Well-designed and systematically maintained registries have been instrumental in advancing the epidemiological understanding of PIDs. These platforms facilitate the development of more effective treatments and personalised care, ultimately leading to improved patient outcomes (19). Despite their importance, national PID registries remain rare in Latin America. Only Paraguay and Venezuela report the existence of a national PID registry.

In other countries, alternative data collection mechanisms exist. Peru and Uruguay maintain registries for rare or genetic diseases, while in El Salvador immunoglobulin treatment registries are maintained at the hospital level. In Argentina, the national patient organisation manages a database comprising 1,143 active patients (as of July 2023). Although these initiatives represent valuable efforts, the absence of comprehensive, standardised national registries remains a major limitation for epidemiological surveillance and strategic planning.

## 4 Discussion

This publication provides the first comprehensive regional overview of the status of living with a PID in Latin America using the PID Life Index. By systematically assessing the implementation of the PID Principles of Care across six domains (diagnosis, treatment availability, universal health coverage, specialised centres, national patient organisations and registries), this analysis shows where countries stand in those domains while revealing gaps that continue to challenge the lives of people living with a PID in Latin America.

### 4.1 Medical education and infrastructure: the adult gap

The findings of the PID Life Index in the region highlight the low diagnostic rate and the acute need to work in improving diagnosis within the adult patients. Despite the availability of basic diagnostic tests in most countries, fewer than 15% of the estimated number of people living with PIDs has been diagnosed in the region. This diagnostic gap stems from structural constraints, including limited access to specialised laboratory testing and insufficient awareness amongst healthcare professionals. Furthermore, it highlights a persistent perception of PIDs as exclusively paediatric conditions, underscoring the urgent need for adult specialists in the region. The transition from paediatric to adult care remains a “salient gap”: without structured transition programs, patients reaching adulthood face a high risk of loss to follow-up and suboptimal disease management.

A major organisational shortcoming identified is the lack of coordinated national networks. In a region characterised by vast territories and big cities, the concentration of expertise in a few urban hubs creates a profound inequity for patients in rural or provincial areas. The lack of formalized healthcare pathways creates significant barriers to care; even when a PID is suspected, the trajectory towards a confirmed diagnosis and specialised treatment remains hindered by fragmented referral networks and prohibitive costs. Strengthening these networks is a must to ensure that the benefits achieved with a diagnosis are not lost due to geographic, organisational barriers, lack of specialists or no communication between centres.

Despite these challenges, there is a clear regional willingness to improve PID care amongst specialists, nurses and patient representatives. A clear example is the meeting organised by the Latin American Society for Primary Immunodeficiencies (LASID) in collaboration with the International Nursing Group for Immunodeficiencies (INGID) and IPOPI in October 2025 in Buenos Aires (Argentina). This meeting highlighted the commitment and willingness of all relevant stakeholders to advance in the diagnosis, treatment and care for people living with PIDs in Latin America.

### 4.2 Availability and access to safe and effective treatments

The PID Life Index illustrates a significant gap in care across the region. While access to vaccines and anti-infectives therapies is relatively stable, there is a systemic failure to provide consistent, lifelong access to essential therapies like immunoglobulins and biological treatments. The fragmented and sometimes very limited coverage of high-cost and long-term treatments exposes patients and their families to financial hardship and clinical instability. This regional landscape presents a challenge for the UHC objective promoted by the WHO, particularly regarding the inclusion of rare and chronic diseases within national health systems. Aligning national and regional policies with the WHO’s objective of “leaving no one behind” requires a focus on integrated health and social models that recognise PIDs as rare and chronic disorders for which care and social protection are needed.

Ig are life-long therapies for a majority of people living with PIDs. Ensuring that these therapies meet high regulatory and medical standards is essential to guarantee patient safety and therapeutic effectiveness. For this reason, these must be subject to rigorous regulatory scrutiny before and after they reach the market. In several countries, patient organisations highlighted this as a major concern during the IPOPI Regional Meeting in Latin America. This reflects the fear of exposure to falsified, substandard or non-optimal therapies when adequate oversight is lacking. Weak or inconsistent regulatory frameworks where no interaction between the authorities and patient organisations exist; may compromise patient trust on the healthcare system and clinical outcomes (22).

Increasing the collection of plasma, the starting material for the development of immunoglobulin replacement therapies is of capital importance. Efforts such as the initiative Plasma4Life, led by UNITAR (the United Nations Institute for Training and Research) promoting capacity building in partner countries such as Argentina, is a key instrument to increasing education around plasma and promote best practices and recommendations to support countries in developing practical solutions to address unmet patient needs.

### 4.3 Patient centricity

Across all principles assessed by the PID Life Index, people living with PIDs in Latin America face not only clinical barriers but also systemic obstacles that directly affect their daily lives, their treatment continuity and long-term health. Delays in diagnosis, uneven access to therapies, limited specialised centres and significant out-of-pocket expenses are not merely structural shortcomings of health systems, they also translate into tangible burdens for patients and their families. Beyond what macro-indicators suggest, the expertise of those living with PIDs is essential in evaluating health services. Their journeys highlight the gap between theoretical policy and the actual clinical experience on the ground.

These findings in this publication reinforce the need to place patient organisations at the centre of policy making and implementation for rare diseases and health and social care in general. The WHO recognises that “the effective and meaningful participation of persons living with a rare disease in decision-making, including through their representative organisations, can strengthen the effectiveness of national, regional and international policy and development programmes related to persons living with a rare disease (23)”. In Latin America, national PID patient organisations already play an essential role in awareness raising, data collection and patient support despite limited resources. Their involvement in health policy discussion, namely on diagnostic pathways, treatment needs, reimbursement frameworks and organisational needs, could substantially improve the alignment of national health systems with the needs of people living with PIDs and contribute to ensuring that no one is left behind in Latin America.

The experience of developing the PID Life Index also highlights the importance of fostering stronger collaboration between patient organisations and healthcare professionals. While the Index relies on information provided by national patient organisations and validated through its established methodology, differences in perspectives may occasionally emerge regarding specific aspects of care provision, such as the availability of specialised centres, referral pathways or access to services. Rather than being viewed solely as discrepancies in reporting, these differences may reflect communication gaps between stakeholders and underline the need for closer dialogue and partnership between patient leaders, clinicians and health authorities. Strengthening these relationships could improve the quality of available information, support more accurate assessments of national realities and ultimately contribute to better care for people living with PIDs.

### 4.4 Public policies and governance for improving care of people living with PIDs

The findings of the PID Life Index also point to the need for stronger public policies and governance mechanisms to address the structural gaps identified across the region for people living with PIDs. Improving PID care requires not only the availability of diagnostic tools and treatments but also sustained and adequate financing, sustained political commitment and coordinated action across health systems. National strategies for rare diseases and PIDs could provide an important framework for defining measurable objectives, clarifying responsibilities amongst health authorities, healthcare providers and funders (public, private or a mixture of both) and ensuring that diagnosis, treatment, follow-up care are addressed across the patient journey. In particular, strengthening referral pathways and networks of specialised centres, establishing or expanding national PID registries, improving access to essential immunoglobulin and other therapies, and developing appropriate reimbursement and procurement policies could contribute to reducing the inequities identified by the Index.

Governance mechanisms should also promote coordination between different levels of the health system and across relevant stakeholders. Ministries of health, regulatory and reimbursement authorities, healthcare professionals, specialised centres and patient organisations all have complementary roles in improving PID care. Regional cooperation may also be valuable, particularly for countries facing limitations in specialised expertise, diagnostic capacity or access to treatments. Sharing good practices, developing common approaches and strengthening regional networks could support countries in addressing challenges that are difficult to overcome through national action alone. An example of multistakeholder collaboration is the Plasma4Life initiative, led by UNITAR (the United Nations Institute for Training and Research) bringing together all key actors to discuss the challenges related to plasma supply with the overall goal to improve access to plasma-derived therapies (such as Ig therapies) for rare disease patients in partner countries such as Argentina. Several limitations of the PID Life Index as a tool should be acknowledged when interpreting the findings of this regional analysis. As previously described, the Index adopts a nation-wide approach that may not fully capture subnational variations due to the wide territories some of the countries in the region have or subnational variations in healthcare organisation, access or coverage policies.

Furthermore, discrepancies may exist between formal policies and their implementation in clinical practices – as shown by some national patient organisations stating the need to fight for their reimbursement rights in court to have the patient’s individual rights respected. This may lead to an overestimation of certain principles, especially in terms of access to diagnosis, treatment and more so universal health coverage.

Data collection also depends on the existence of an active national PID patient organisation or a recognised medical advisor to the patient organisation, which may influence both country coverage and the completeness of the information provided. Finally, responses may be subject to individual or organisational bias, influenced by respondents’ level of knowledge, experience and good faith.

## 5 Conclusion

Despite these limitations, the data presented provide a robust and informative snapshot of the status of PID care in Latin America and the Caribbean across the six Principles of Care for PID. The consistency of findings across countries, especially regarding low diagnosis, limited implementation of newborn screening for SCID, uneven access to immunoglobulin replacement therapies, gaps in universal health coverage, and the absence of a coordinated network of specialised centres or of registries, suggests that the challenges identified are structural to the region.

Addressing these structural gaps will require sustained public policies and governance actions, including stronger national strategies for PIDs and rare diseases, adequate and equitable financing, coordinated referral and specialised care networks, appropriate treatment coverage or reimbursement and procurement mechanisms, stronger regulatory oversight and collaboration, and meaningful involvement of patient organisations in policy development and implementation. Regional collaboration and the sharing of existing good practices may further support countries in strengthening their capacity to provide equitable and sustainable PID care.

As the PID Life Index continues to evolve as a longitudinal process, expansion to additional countries and iterative refinement of the questionnaire are expected to further reduce bias and strengthen data robustness. Future research should build on this baseline by examining changes in PID care indicators over time and by exploring the relationship between policy implementation and improvements in patient access and outcomes. Further studies could also investigate the implementation of referral pathways and transition from paediatric to adult care, the impact of reimbursement and procurement policies on treatment continuity, and the perspectives and experiences of people living with PIDs. Such evidence would help identify which policy and health-system interventions are most effective reducing the gaps identified in this study. This publication therefore represents a meaningful baseline for monitoring progress, informing policy dialogue, and guiding targeted interventions to improve equitable access to PID care in the region.

## Data Availability

The original contributions presented in the study are included in the article/supplementary material. Further inquiries can be directed to the corresponding author.
